# Influence of Epididymal Anomalies and Development of Processus Vaginalis on Testicular Migration And Cryptorchidism. A Narrative Review

**DOI:** 10.1590/S1677-5538.IBJU.2026.9905

**Published:** 2026-04-25

**Authors:** Feridun C. Tanyel, Luciano A. Favorito

**Affiliations:** 1 Turkish Academy of Sciences Ankara Türkiye Turkish Academy of Sciences, Ankara, Türkiye; 2 Universidade do Estado do Rio de Janeiro Unidade de Pesquisa Urogenital Rio de janeiro RJ Brasil Unidade de Pesquisa Urogenital - Universidade do Estado do Rio de Janeiro – UERJ, Rio de janeiro, RJ, Brasil

**Keywords:** Cryptorchidism, Epidydimal anomalies, Infertility, Testicular Hormones

## Abstract

The objective of this narrative review is to show the most relevant aspects of the structure of epidydimal anomalies (EAs) and processus vaginalis (PV) during testicular migration and in patients with undescended testis. Smooth and striated muscles develop around the processus vaginalis (PV) within the gubernaculum during fetal life. The testis migrates through the PV by the means of peristaltic activity generated by those muscles. After migration is completed, the smooth muscle undergoes programmed cell death, resulting in obliteration of PV and leaving only the dartos muscle. Initiation of programmed cell death requires a shift in autonomic balance characterized by a decrease in sympathetic tone and a concomitant increase in parasympathetic tone. The shift represents a critical step in the migration process. If the shift occurs prematurely, smooth muscle mass decreases before migration is completed, potentially preventing adequate propulsion of the testis and resulting in undescended testis. Premature shift is persistent. Persistence of this shift links the clinical features associated with failed migration. Conversely, if the shift fails to occur or is insufficient, smooth muscle persists and inhibits obliteration of the PV, leading to inguinal hernia or hydrocele depending on the remaining amount of smooth muscle. Epididymal disjunctions anomalies and epididymis atresia could be associated with infertility and are very usual in undescended testis. We can divided EAs in 3 groups: normal variant, disjunction anomalies and epididymis atresia. In the normal variant the epididymis is attached to the testis at the head and tail and totally attached to the testis. The transient presence of SM, its programmed elimination, and regulation by autonomic tone together offer a coherent model that integrates developmental, physiological, and clinical observations. EAs are frequently found in cryptorchidism. Knowledge of anomalies associated with cryptorchidism is relevant in clinical practice, both to prevent accidents during orchidopexy and to counsel and predict infertility in the future, such as in cases of epididymis atresia and total disjunction between the testis and the epididymis.

## INTRODUCTION

Undescended testis can be associated with several anatomical anomalies, but epididymal anomalies (EAs) and patency of the processus vaginalis (PV) are among the most frequent with a very variable incidence in the literature: from 36 to 79% (1-4). Knowledge of EA associated to cryptorchidism is relevant in clinical practice to prevent accidents during orchidopexy and to counsel and predict infertility in the future, such as in cases of epididymis atresia, disjunction of epididymal head and total disjunction between the testis and the epididymis (5, 6).

Some studies show an association of the patency of the PV with EAs and cryptorchidism, suggesting that there is a common stimulus, probably androgenic, participating in the epididymis development, testicular migration and closure of the PV (3, 7). The objective of this review is to show the most relevant aspects of the structure of EAs and PV during testicular migration and in patients with undescended testis.

## PROCESSUS VAGINALIS AND TESTICULAR MIGRATION

Despite centuries of attempts to explain testicular migration, none has provided a fully satisfactory account. A paradigm shift offers a more coherent mechanistic explanation (8, 9). Migration of the testis can be conceptualized as work, defined as force multiplied by displacement. Hormones alone do not generate force; therefore, migration requires a structure capable of producing force under hormonal modulation. Failure of migration results in an undescended testis, and any explanation must integrate both the mechanism of descent and the reason for its inhibition.

The processus vaginalis (PV) serves as the pathway for testicular migration. Following descent, the PV should undergo obliteration; failure of this process leads to inguinal hernia or hydrocele. Undescended testis is associated with vaso-epididymal anomalies, disrupted mini-puberty, reduced fertility, and increased risk of malignancy. A testis that has initially descended may later retract or ascend (10). A comprehensive mechanism should therefore provide a unified explanation for these associated observations.

### Patent processus vaginalis and smooth muscle persistence

Sacs obtained from boys with inguinal hernia or hydrocele consistently present smooth muscle (SM) and myofibroblasts beneath the mesothelial layer, whereas obliterated PV lacks these structures (11-13). Multiple independent studies have confirmed that persistence of SM inhibits obliteration of PV (14-23).

Residual SM not only prevents obliteration but may also contribute to clinical complications. For example, persistence of SM may be associated with an increased risk of incarceration in preterm infants and elevated hydrocele pressures (24-26).

### Muscular development in the gubernaculum

The PV develops into the gubernaculum. Youssef and Raslan have reported the development of SM in gubernaculum, which disappears after descent (27).

Our studies have demonstrated that striated muscle fibers and myofibroblasts appear within the gubernaculum at 22 weeks of gestation, whereas SM develops around the PV at approximately the 27th week. Cremaster muscle may transdifferentiate from the vascular SM. Myofibroblasts appear to represent an intermediate stage within the differentiation- dedifferentiation continuum of SM (28).

The gubernaculum that is described as primitive mesenchymal tissue ceases to exist after development of muscles. We have speculated that INSL3 takes part in myogenesis (8). Yuan et al. subsequently provided support for this proposal (29).

### The physiological role of smooth muscle

The transient presence of SM around the PV serves a specific physiological role: propulsion of the testis during migration. This propulsion explains both successful descent and the risk of intrauterine torsion (8-10). Once migration is completed, the SM undergoes programmed cell death, leaving only the dartos muscle. This interpretation provides a framework that integrates the mechanical, developmental, and pathological aspects of testicular descent.

### Programmed cell death of smooth muscle depends on shift in autonomic tonus

After a structure completes its function, it disappears through programmed cell death (PCD). PCD involves both intrinsic and extrinsic pathways. The intrinsic pathway involves calcium signaling that increases cytosolic calcium and promotes mitochondrial calcium loading, leading to opening of permeability transition pores (9).

Maintenance of SM appears to depend on sympathetic tone, whereas activation of the inositol 1,4,5-trisphosphate pathway releases calcium from the intracellular stores and initiate PCD.

These observations suggest that a transient decrease in sympathetic tone combined with an increase in parasympathetic tone may represent the physiological mechanism responsible for PV obliteration.

Silencing of the central catecholaminergic activity may contribute to this shift (9). The key to inguinal hernia, hydrocele and undescended testis lie in this shift of autonomic tone. Deviations in the timing, intensity, or duration of this may lead to pathological outcomes. Because sympathetic tone is sexually dimorphic, this shift may occur more readily in females.

### Early shift inhibits migration whereas ineffective shift results in inguinal hernia or hydrocele

If the autonomic shift occurs before testicular migration is completed, premature PCD may reduce the amount of SM surrounding the PV. Remaining SM may not suffice to propel the testis. Minimal SM content results in an undescended testis located at a higher position (8).

If the intensity or duration of shift in autonomic tone is not as effective as required, SM does not undergo PCD and persists (30-32). Persistence of SM inhibits the obliteration of PV, resulting in inguinal hernia or hydrocele depending on the remaining amount of SM (Figure-1) (9,10).

**Figure 1:**
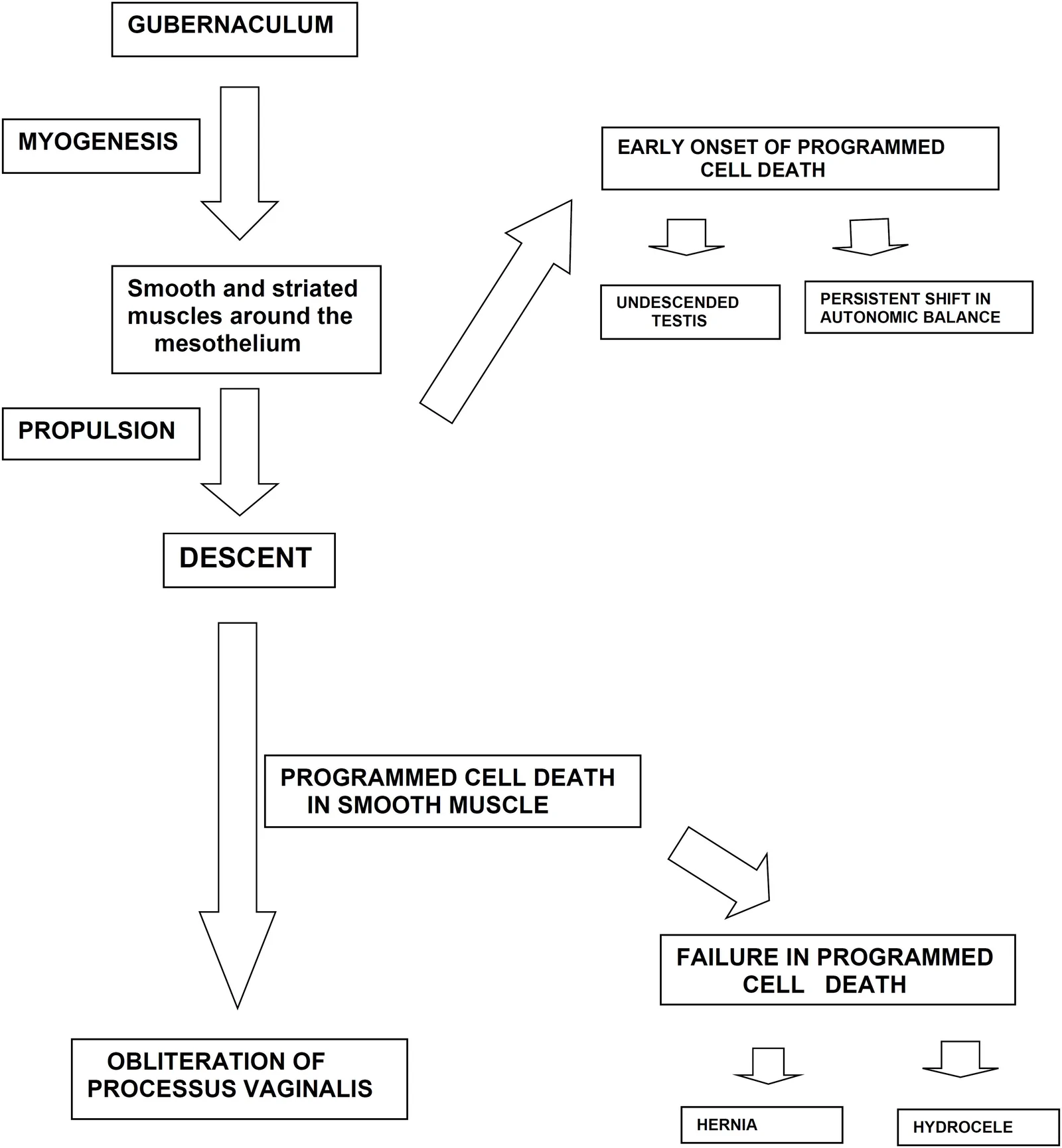
Schematic summary of Testicular Migration.

### Persistence of autonomic imbalance forms basis for the associated features encountered in undescended testis

Sacs, cremaster muscles and heart rate variability analysis from boys with undescended testis reveal evidence of defective autonomic innervation via increase in parasympathetic but decrease in sympathetic tones (33-40). Persistence of shift in autonomic tone provides basis for the associated occurrences such as decrease in fertility, increase in risk of malignancy, vaso-epididymal anomalies, disrupted mini-puberty and retractility and ascent.

While parasympathetic tone acts through generating phospholipase C, sympathetic tone acts by generating cyclic adenosine monophosphate (cAMP). Continuous stimulation of phospholipase C pathway by phorbol esters results in the development of tumors. On the other hand, the cAMP pathway is important for spermatogenesis. Differences in signaling pathways with dominance of phospholipase C and decrease in cAMP pathways explain the increase in the risk of malignancy and decrease in fertility (Figure-2) (8, 9). Persistence of signaling towards inducing PCD may also play a role in the reduction of number of germ cells associated with undescended testes.

**Figure 2:**
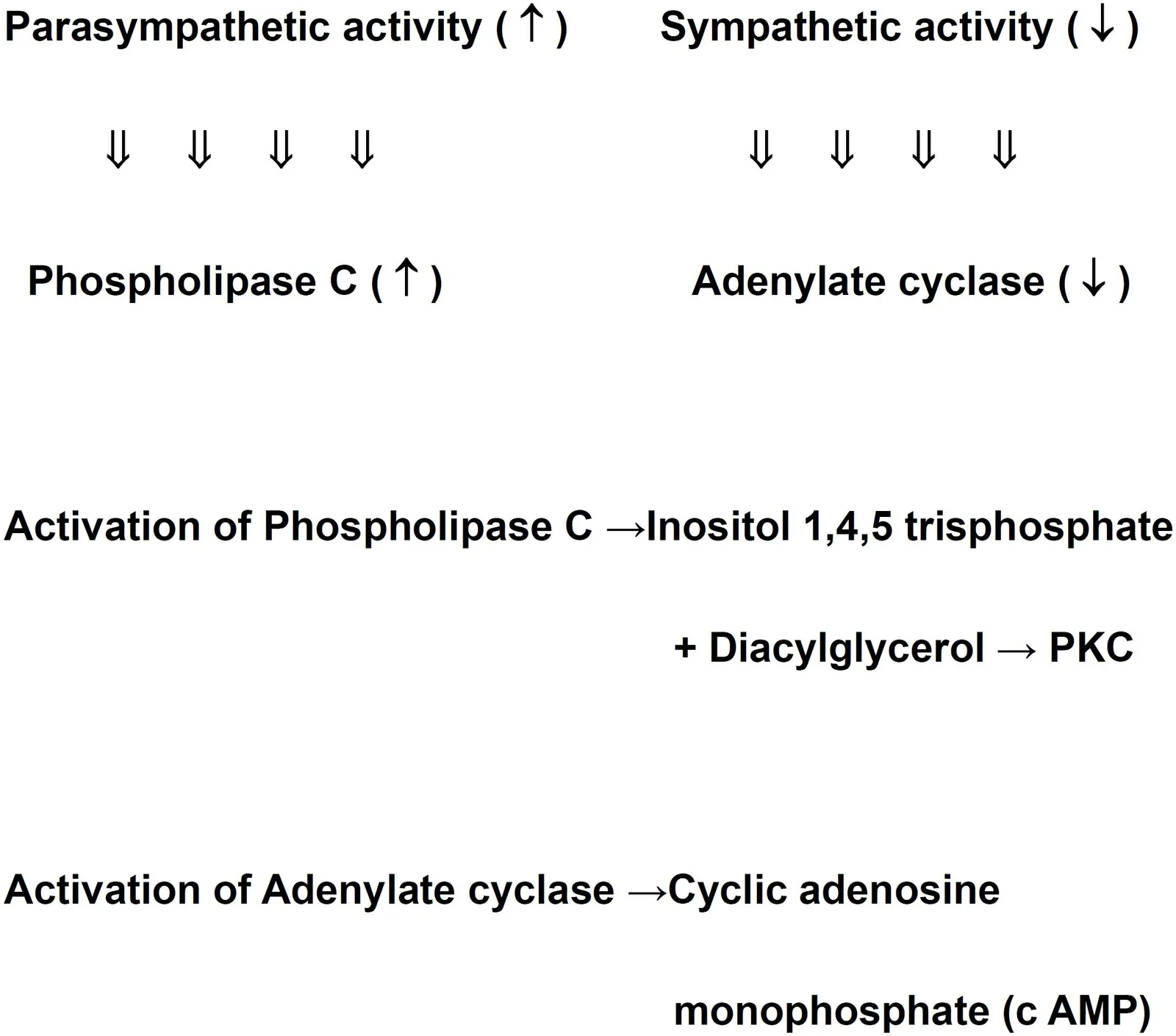
Signaling during persistent shift in autonomic tonus explains the increase in risk of malignancy and decrease in fertility.

The gubernaculum also supports the muscular layer of vaso- epididymal structures. Signals towards inducing programmed cell death in SM explains both the reason of vaso-epididymal anomalies and their association with undescended testis (9).

Central catecholaminergic activity also takes place in the regulation of hypothalamic- pituitary- gonadal axis. Since catecholamines take place in the control of pulsatile release of gonadotropin releasing hormone, silencing of central catecholaminergic activity may also affect the hypothalamic- pituitary- gonadal axis (9, 41). On the other hand, testosterone release is also regulated by an efferent neural pathway from the hypothalamus to the testes under the influence of catecholamines (42). Chemical sympathectomy reduces the concentration of testosterone (43). The silencing of central catecholaminergic activity satisfactorily explains the decrease in release of testosterone from the Leydig cells in response to hCG.

Hypogonadism may result from central catecholaminergic changes directed to shift the autonomic tone. Disrupted mini puberty does not provide basis to inhibit descent but shares the persistent shift in autonomic tone (44).

A migrated testis may subsequently retract or ascend. Retractile testis has traditionally been explained by a hyperactive cremaster reflex. However, the cremaster reflex cannot be repeatedly induced without a refractory period due to desensitization. Induction of an already induced superficial reflex is not possible until reflex arc is completed. If the retraction results from a reflex arc, the testis should move up and down and stay in the scrotum after desensitization. However, there are no such testes (45- 47). The increase in parasympathetic but decrease in sympathetic tones increases cytosolic calcium. Increase in cytosolic calcium contracts a striated muscle. Increased contractility and contracture define the place in the spectrum as retraction or ascent. Cremaster muscles associated with retractile testes share similar alterations as in patients with undescended testis (48, 49). Retraction or ascent does not reflect a hyperactive reflex but reflects the increase of cytosolic calcium in cremaster muscles due to shift in autonomic tones.

## EPIDIDYMIS AND TESTICULAR MIGRATION

Testicular migration is a complex process mediated by endocrine and mechanical factors. The integrity of the axis between the testis, hypothalamus and pituitary, which regulates testosterone production, is important for this process.

Cryptorchidism is a common event in pathologies on this axis, such as hypogonadotropic hypogonadism and 5-alpha reductase deficiency (50). Testosterone appears to play an active role in testicular migration, inducing the development of important structures for testicular migration such as the vaginal process, the vas deferens, the epididymis, the inguinal canal and the scrotum. Another mechanism of action of testosterone would be through stimulation of the genitofemoral nerve, which would induce the production of calcitonin gene-related peptide (CGRP) that acts by stimulating the development of the testicular gubernaculum (51).

Fetal and placental gonadotropins are also implicated in the process of testicular migration. These substances act by stimulating the production of testicular androgens, which induce the growth and development of the vas deferens, the epididymis, the vaginal process and the gubernaculum, itself (52). It is well known that the treatment of cryptorchidism with gonadotropins induces testicular migration at levels ranging from 25 to 55% of cases (53).

Another endocrine substance involved in testicular migration would be descendin. This androgen-independent secreted substance in the testis would play an important role in the growth of the gubernaculum mesenchymal cells. The gubernaculum would therefore be one of the fetal structures implicated in testicular migration, most modified by hormonal action.

Problems with the hypothalamus-pituitary axis are associated with cryptorchidism, which explains the high incidence of this anomaly in patients with Prader-Willi syndrome, Kallman syndrome, pituitary hypoplasia and anencephaly.

Analysis of the nerves of the cremaster muscle of patients with retractile testis also revealed significant differences. These patients had a lower concentration of nerve fibers compared to those with inguinal hernia, but no differences in relation to the group with cryptorchidism. The innervation of the cremaster muscle and the gubernaculum testis originates in the genitofemoral nerve (53). This nerve, through androgenic regulation and the influence of the calcitonin gene-related peptide (CGRP), plays an important role during the testicular migration process, by neuromuscular junction stimulation of the rhythmic contractions of the cremaster muscle during the fetal period (52).

The epididymis is anatomically connected to the gubernaculum, which in turn is attached to the testicle and scrotum (Figure-3). The gubernaculum seems to be the most important anatomical structure in the testicular migration process, by means of contraction and shortening, thus imposing traction strength on the testis and facilitates the transition of the testis by through the inguinal canal (53, 54) (Figure-3).

**Figure 3:**
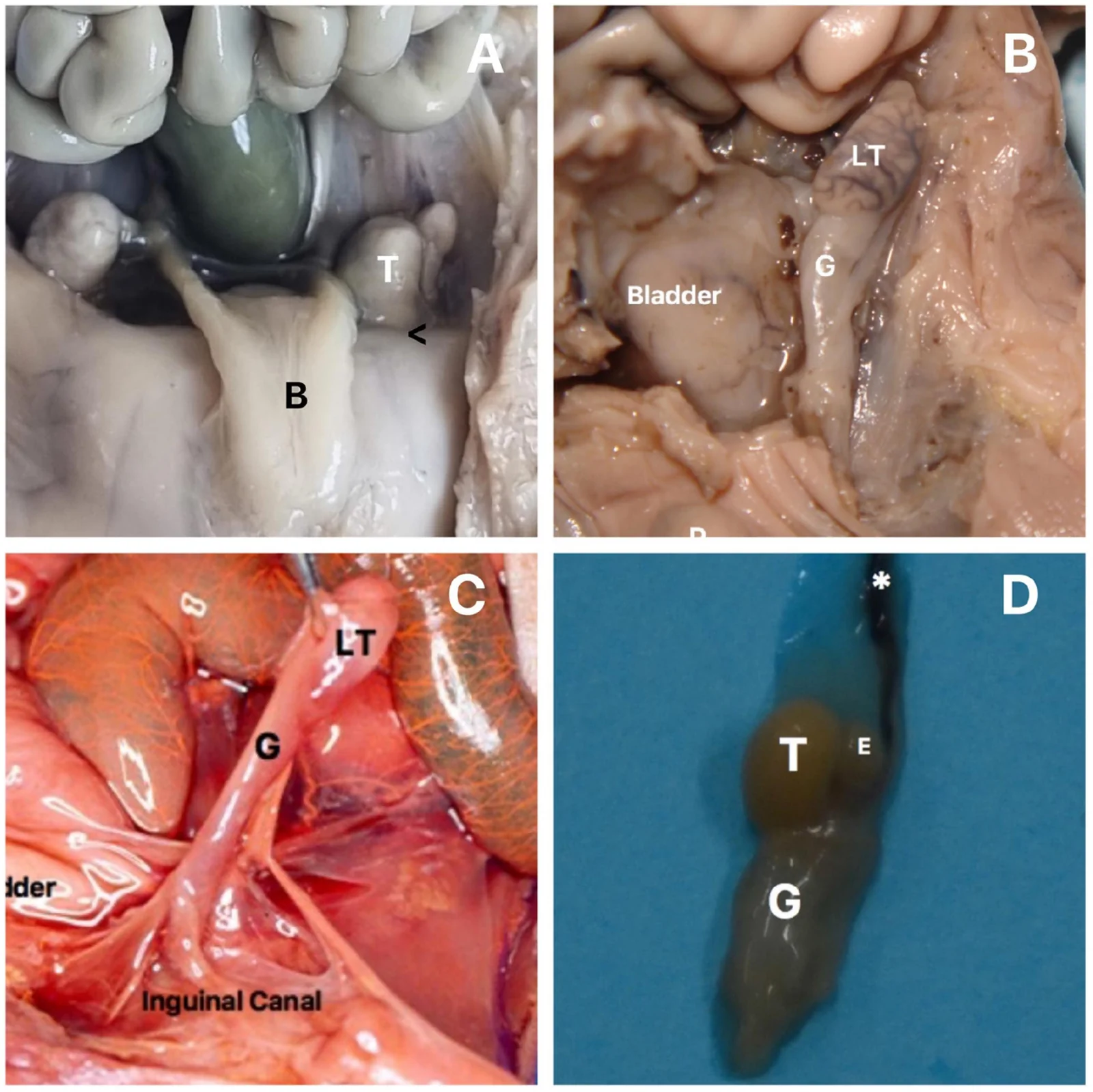
Testicular migration and gubernaculum.

A) The figure shows a fetus with 18 weeks post-conception (WPC) showing the testis (T) beginning its transition in internal inguinal ring (<), B- bladder; B) The figure shows the dissection of the inguinal canal in a fetus with 17WPC. We can observe the gubernaculum (G) attachments with the left testis (LT) and epididymis proximally and with the inguinal region distally; C) In this figure we can observe the dissection of the gubernaculum (G) in a fresh human fetus with 18 WPC, LT – left testis, C- colon and D) The testis (T), epididymis (E) and gubernaculum (G) were removed from a fetus with 20WPC, observe the testicular vessels (*) in proximal position of the testis

The growth of the vaginal process divides the gubernaculum into three parts: (a) the main gubernaculum, which corresponds to the portion covered by the visceral layer of the peritoneum of the vaginal process; (b) the vaginal gubernaculum, which corresponds to the portion that externally surrounds the parietal portion of the vaginal process, and (c) the infra-vaginal gubernaculum, corresponding to the caudal region of the gubernaculum, which has not been invaded by the vaginal process (55).

Both the gubernaculum and vaginal process change in harmony during testicular migration. The maintenance of this undifferentiated mesenchyme along the inguinal canal and scrotum is essential for the downward extension of the vaginal process to occur, during which it follows the pathway created by dilation of the gubernaculum, forming the canal through which the testis will reach the scrotum (53, 55).

An interesting and controversial theory suggests that the epididymis would be one of the responsible for testicular migration, through its peristaltic and secretory activity in the second gestational trimester (56). This theory would explain some cases of cryptorchidism, where the epididymis is separated and located more inferiorly than the testis (57). Several observations support the theory described above. The epididymis precedes the testicle in the scrotum; the epididymis is in a privileged position to influence testicular migration, as it is anatomically connected to the gubernaculum, which in turn is attached to the testicle and scrotum. Morphological and functional changes occur in epididymis at the time of migration in certain animal species (58, 59).

## EPIDIDYMAL ANOMALIES AND UNDESCENDED TESTIS

There are several classifications to analyze the relations between the testis and epididymis (60, 61). We can divide this relationship in 3 groups: normal variant, disjunction anomalies and epididymis atresia. In the normal variant the epididymis is attached to the testis at the head and tail (Figure-4) and totally attached to the testis (Figure-5).

**Figure 4:**
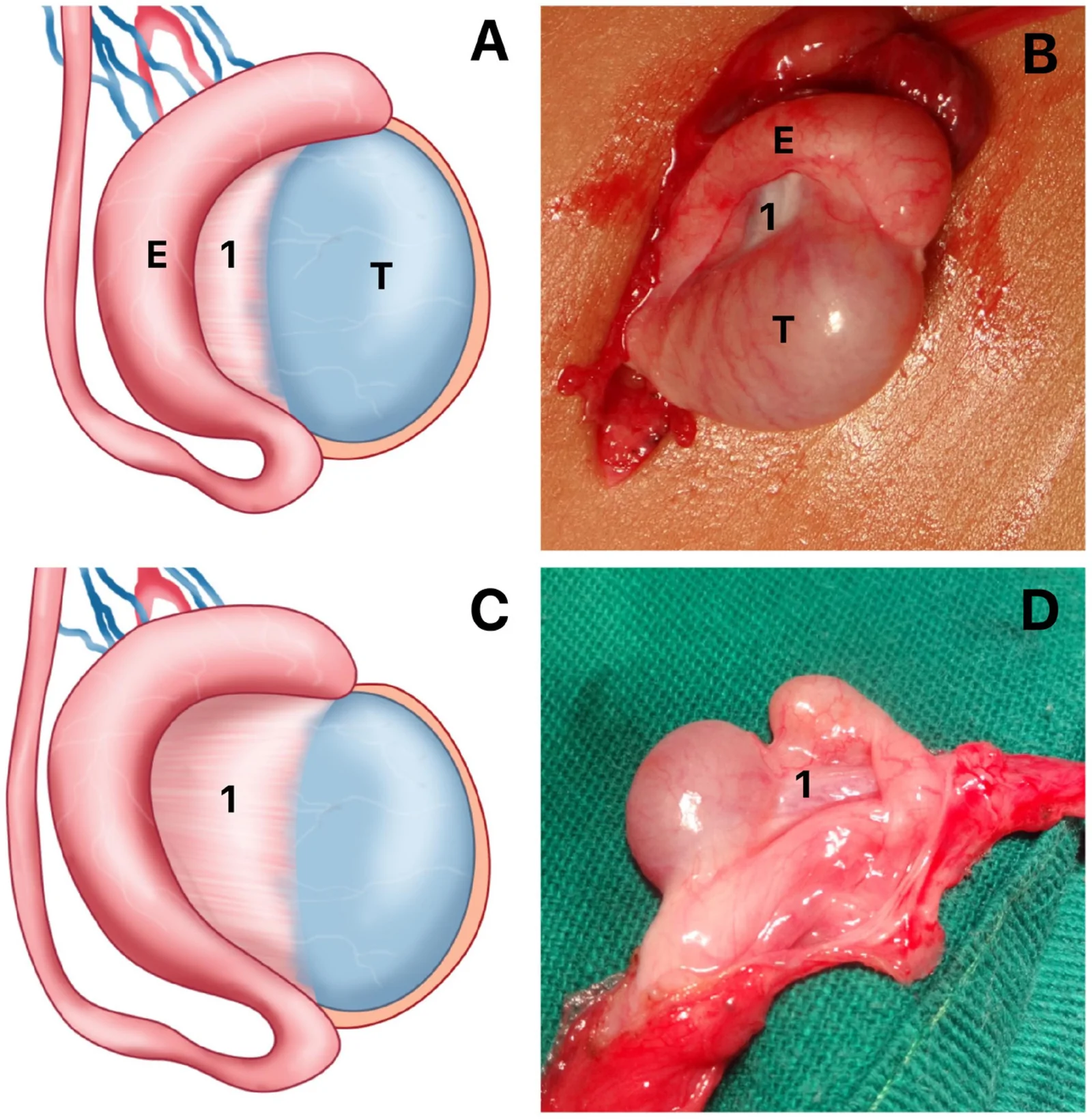
Relationship between testis and epididymis - Normal variant.

A) Schematic drawing showing a normal variant of epidydimal anatomy - the epididymis (E) is attached to the testis (T) at the head and tail, and we can observe the mesorchium (1); B) We can observe in a undescended testis patient with 3 years old a normal variant of the epididymis (E) – Epididymis is attached to the testis (T) at the head and tail, 1- mesorchium; C) Schematic drawing showing an elongated epididymis with a long mesorchium (1) and D) The same situation in a undescended testis patient with 4 years-old, an elongated epididymis with long mesorchium.

**Figure 5:**
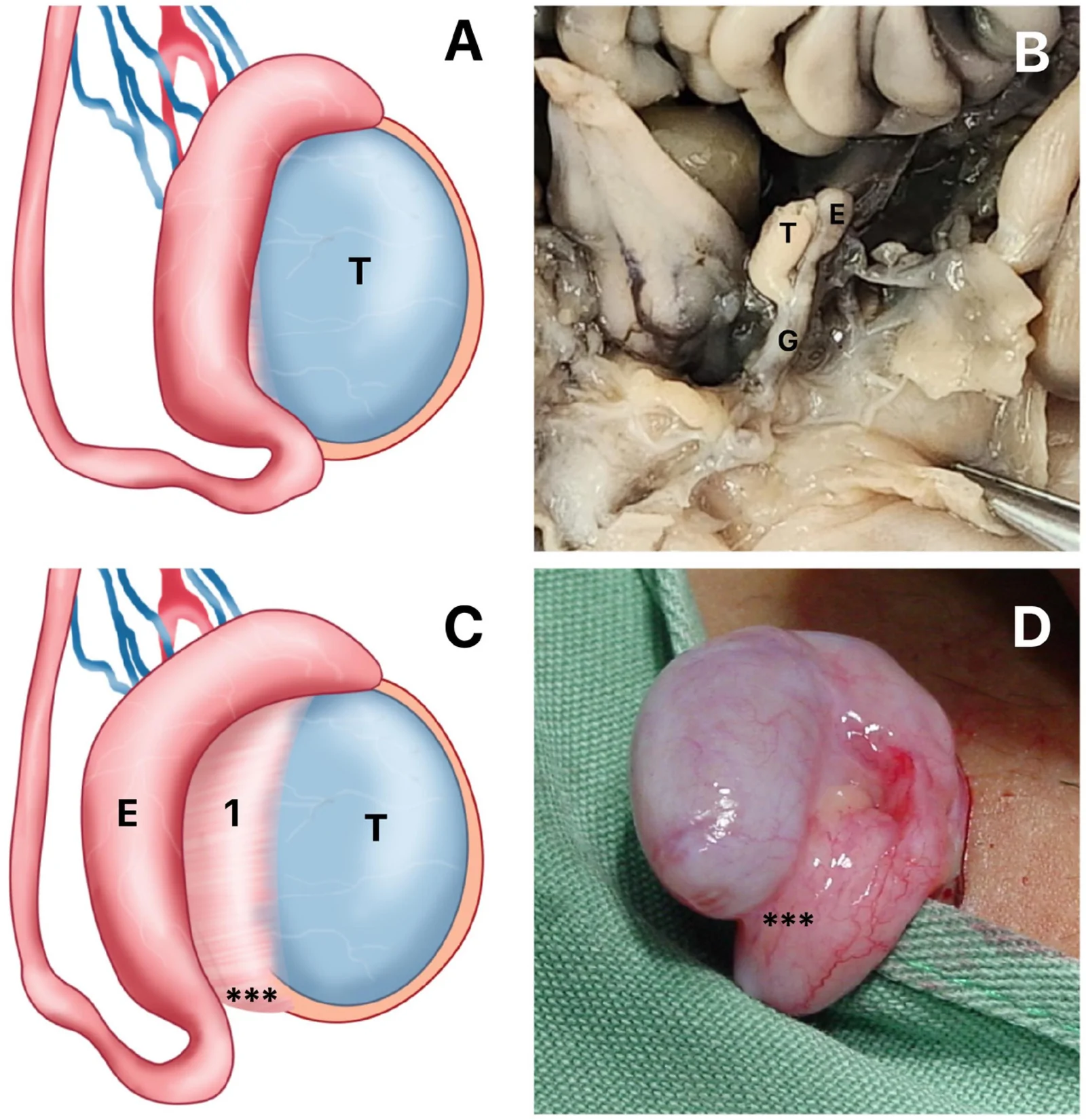
Testis attached to epidydimis and Epididymal tail disjunction.

A) Schematic drawing showing a normal variant of the epididymis anatomy - the epididymis is attached to the testis (T) at the head and tail; B) We can observe in a fetus with 16 weeks post-conception a normal variant of the epididymis anatomy - the epididymis (E) is attached to the testis (T), G- gubernaculum; C) Schematic drawing of a epididymal anomaly – disjunction of the epididymal tail (***), T- testis, E- epididymis and 1 – mesorchium and D) We can observe a disjunction anomaly of the epididymal tail (***) in a undescended testis patient with 1 year-old.

We can observe three types of epididymal disjunction anomalies: Epididymis attached to the testis only at the head (Figure-5); epididymis attached to the testis only at the tail (Figure-6) and no visible connection between the testis and epididymis (Figure-6). The EAs with head disjunction and total disjunction are associated to infertility (62). We can observe example of epididymal atresia in Figure-7.

**Figure 6:**
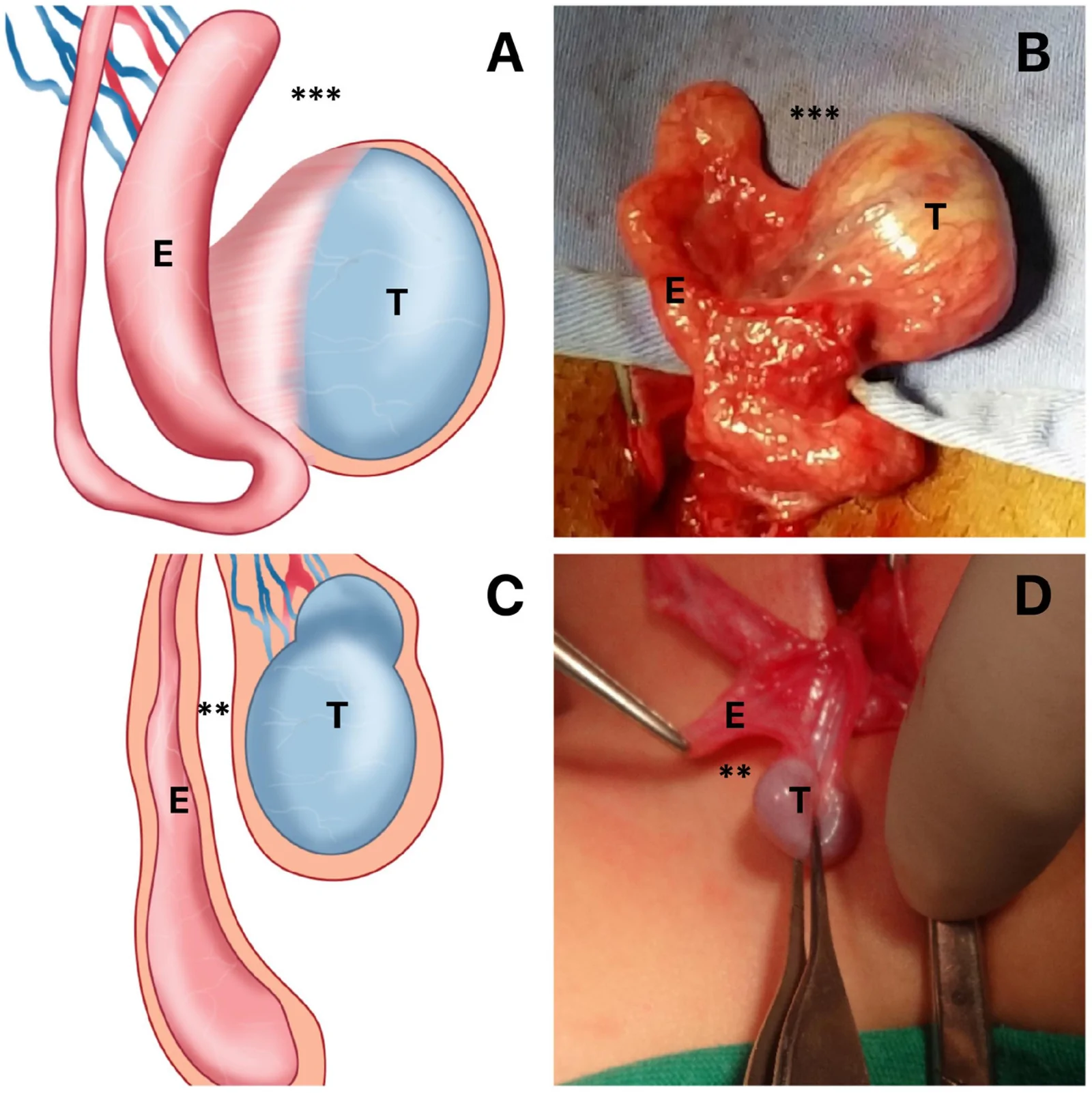
Epididymal anomalies.

A) Schematic drawing showing the epididymal anomalies of head disjunction (***), T- testis, E- epididymis; B) We can observe in a undescended testis patient with 4 years-old the epididymal anomalies of head disjunction (***),T- testis, E- epididymis; C) Schematic drawing showing the total disjunction (***) between the testis (T) and epididymis (E) and D) We can observe in a undescended testis in a patient with 2 years-old the total disjunction (***) between the testis (T) and epididymis (E

**Figure 7:**
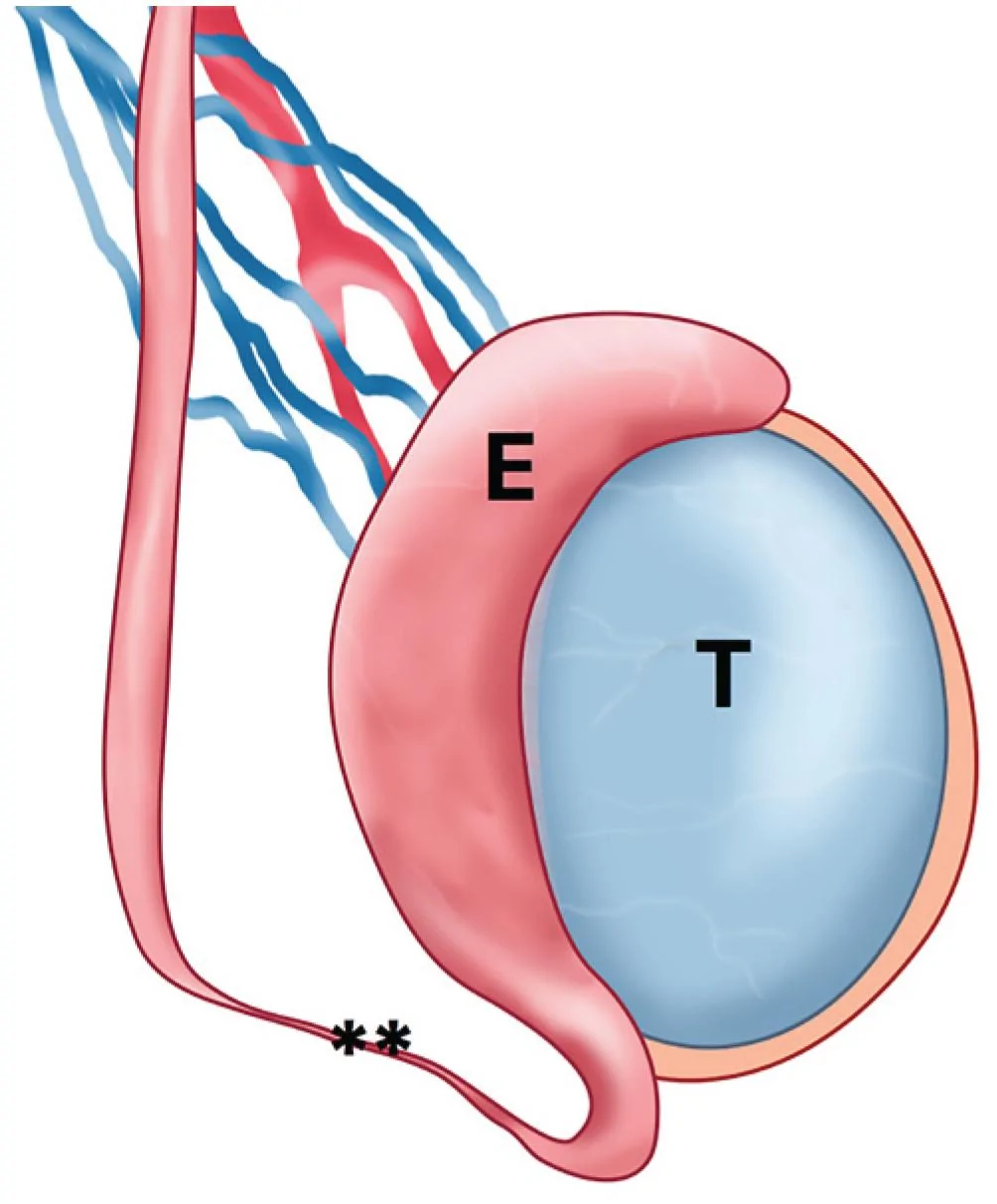
Schematic drawing showing the epididymal atresia.

T- testis, E-epididymis and ** - atresia of the epididymal tail.

Mollaeian (1) studied 652 undescended testes and observed epididymal disjunction in 16.87% of the cases. Caterino (63) in a study with 895 patients and Kim (2) in a study with 110 patients reports EAs, respectively, in 96% and 65% - a very high index and probably this high number is associated to a classification that considers normal relationships between testis and epididymis as an anomaly. In a previous study of our group, we observed EAs in 43.7% of the cases; the disjunction of epididymal tail was observed in 25,3%, and disjunctions of epididymal head and total disjunction of epididymis were observed in 18.4% of the cases (62).

The discordant number of EAs associated to undescended testis in several studies can be justified by the lack of definition of the normal pattern of the anatomy of the epididymis in several studies (60, 61). The major risk of infertility in patients with undescended testis may be result from obstruction associated to epididymal disjunctions anomalies and epididymis atresia (64, 65). The disjunction of epididymal head and total disjunction of epididymis are usual in undescended testes and more frequent in testes situated in high position and with patency of the PV (64, 65). The PV is usually obliterated after the end of the testicular migration (66-68). The patency of the PV in patients with cryptorchidism ranges from 21.3 to 81.3% (2, 66).

The epididymal anomalies of disjunction can be explained by the difference in the embryological origin of the epididymis from the mesonephric duct or by changes in the vascularization during the development of the mesonephric tubules and their fusion with the testicular tubules (69). So we suggest the classification of the epididymal anomalies in disjunctions anomalies, epididymal atresia and elongated epididymis.

## EPIDIDYMAL ANOMALIES AND TESTICULAR TORSION

The testicular torsion is an anomaly resulting from changes in the implantation of the tunica vaginalis or epididymal disjunction (70). Normally, the testis is united to the tunica vaginalis, and if the tunica is implanted too high, the testis can present excessive mobility (bell clapper testis) (70). Mesorchium is the ligament that unites the testis to the epididymis (58). In cases of epididymal disjunction or elongated epididymis, conditions that are highly frequent in cryptorchism (69, 71, 72) the mesorchium is long and can contribute to the testicular torsion (58) (Figures 5 and 6).

The type of testicular torsion was classified in three groups according to the anatomy of the tunica vaginalis and the relationship between testis and epididymis (73) (Figure-8): Group A – bell clapper testicular deformity (leading to intravaginal torsion); Group B – torsion of spermatic cord (leading to extravaginal torsion) and Group C – torsion due to long mesorchium (Figure-8). Bell clapper deformity (intravaginal torsion) is the most commonly found type of testicular torsion (80%).

**Figure 8:**
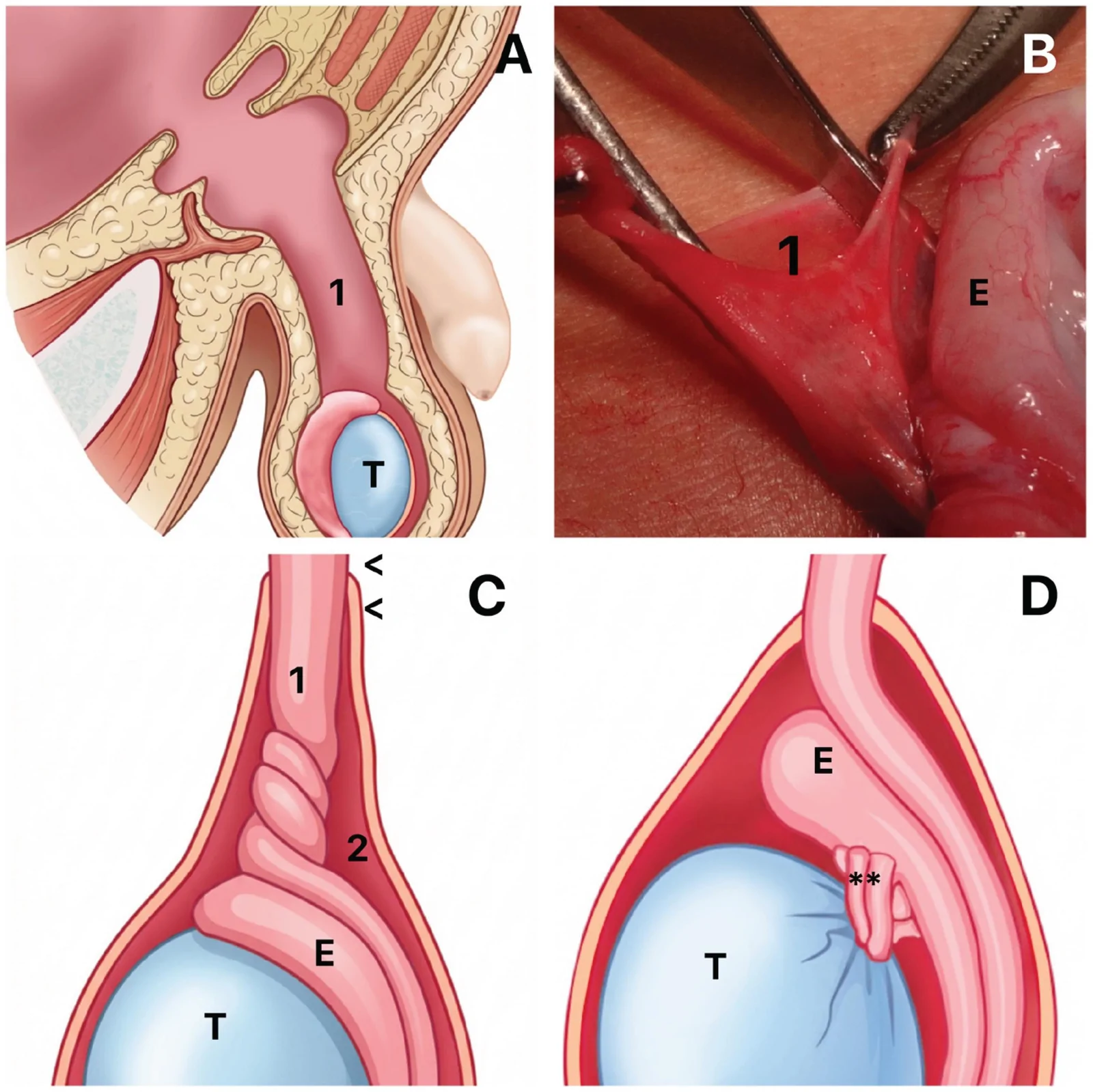
Patency of Processus vaginalis.

A) Schematic drawing showing the patency of Processus vaginalis (1), T- testis; B) We can observe in a undescended testis patient with 6 years-old the patency of processus vaginalis (1), E- Epididymis; C) schematic drawing showing the high implantation (<<) of the tunica vaginalis (2) in spermatic vessels (1): the bell clapper testicular deformity (leading to intravaginal torsion), T- testis, E- epididymis and D) Schematic drawing showing the testicular torsion due to long mesorchium (**), T- testis, E- epididymis.

Testes present a normal layering of tunica vaginalis. They are involved by this structure on both sides and on their upper portion. The posterior region of testis is not covered by tunica vaginalis. United to the lower pole region of the testis and the epididymal tail, there is the testicular gubernaculum or its remnant, the testicular ligament, which is covered by tunica vaginalis only in its anterior and lateral portions (58). Intravaginal testicular torsion does not occur in testes presenting normal anatomy as described above, because the posterior testicular segment is firmly united to the scrotum, preventing the organ from moving (58, 70).

Normal anatomy of tunica vaginalis or epididymis at the side contralateral to the torsion is very rare and anatomic anomalies occur bilaterally in the vast majority of cases. These findings stress the need for bilateral orchiopexy in cases of testicular torsion (73). Cases of torsion due to long mesorchium most often occur as a consequence of anomalies of epididymal disjunction or elongated epididymis, conditions that are highly frequent in cryptorchism (58). Of the 8 cases with long mesorchium, 3 (37.5%) had cryptorchid testes.

Approximately 20% of cases of testicular torsion occur in patients with cryptorchism (58). Epididymal anomalies associated with long mesorchium are frequent in patients with cryptorchism, with an incidence ranging from 36 to 72% (71) and rare in individuals with topic testes (less than 4%) (60). Due to these changes in mesorchial region, the possibility of testicular torsion must be considered in cryptorchid patients presenting acute scrotal or inguinal pain.

The intravaginal torsion (bell clapper tunica vaginalis) is the most frequent type of torsion, and torsion due to long mesorchium is associated with cryptorchism. The most frequently found anatomical relation between testis and epididymis in the study group was type I (epididymis united to the testis by its head and tail). Historically the bell-clapper deformity is the only anatomical risk factor for the intravaginal torsion but the epididymal dysjunction, despite rare, also play a hole in the pathology. In approximate 90% of the time, a vestigial of Müllerian duct keeps present attached to the upper pole of the testis and is called appendix testis or hydatid of Morgani. This appendix can also twist during lifetime, mimics the testicular torsion clinical presentation and sometimes also requires surgical treatment (74, 75).

## CONCLUSIONS

Evaluating testicular migration within this mechanistic framework provides a unified explanation for both successful descent and its associated disorders. The transient presence of SM, its programmed elimination, and regulation by autonomic tone together offer a coherent model that integrates developmental, physiological, and clinical observations.

EAs are frequently found in cryptorchidism. Knowledge of anomalies associated with cryptorchidism is relevant in clinical practice, both to prevent accidents during orchidopexy and to counsel and predict infertility in the future, such as in cases of epididymis atresia and total disjunction between the testis and the epididymis.

## Data Availability

All data generated or analysed during this study are included in this published article
